# Enhancement of Biodegradation of Palm Oil Mill Effluents by Local Isolated Microorganisms

**DOI:** 10.1155/2014/727049

**Published:** 2014-08-12

**Authors:** Mohammadreza Soleimaninanadegani, Soheila Manshad

**Affiliations:** Department of Biotechnology and Medical Engineering, Faculty of Biosciences and Medical Engineering, Universiti Teknologi Malaysia (UTM), 81310 Skudai, Johor, Malaysia

## Abstract

This study was designed to investigate the microorganisms associated with palm oil mill effluent (POME) in Johor Bahru state, Malaysia. Biodegradation of palm oil mill effluents (POME) was conducted to measure the discarded POME based on physicochemical quality. The bacteria that were isolated are *Micrococcus* species, *Bacillus* species, *Pseudomonas* species, and *Staphylococcus aureus*, while the fungi that were isolated are *Aspergillus niger*, *Aspergillus fumigatus*, *Candida* species, *Fusarium* species, *Mucor* species, and *Penicillium* species. The autoclaved and unautoclaved raw POME samples were incubated for 7 days and the activities of the microorganisms were observed each 12 hours. The supernatants of the digested POME were investigated for the removal of chemical oxygen demand (COD), color (ADMI), and biochemical oxygen demand (BOD) at the end of each digestion cycle. The results showed that the unautoclaved raw POME sample degraded better than the inoculated POME sample and this suggests that the microorganisms that are indigenous in the POME are more effective than the introduced microorganisms. This result, however, indicates the prospect of isolating indigenous microorganisms in the POME for effective biodegradation of POME. Moreover, the effective treatment of POME yields useful products such as reduction of BOD, COD, and color.

## 1. Introduction

Palm oil industry has become one of the main Agro-industries in Malaysia. Palm oil mills release POME in colossal amounts with its attendant polluting impending. POME has unfavourable environmental ramifications effects including land and aquatic ecosystem contamination and loss of biodiversity and increase in COD and BOD in environment [1, 2]. Today, the penetration of palm oil mill has been considered due to its effluents into the waterways and ecosystems remaining a meticulous concern towards the food chain interference and water consumption [3]. This can cause considerable environmental problems if discharged without effective treatment by polluting land and effectively suffocating other aquatic life [4]. Thus, palm oil mills are required to treat their POME prior to discharging it into rivers and streams. In the process of palm oil milling, POME is mainly generated from sterilization and clarification of palm oil in which a large amount of steam and hot water are used [5]. POME is a thick brownish liquid that is compiled with high concentrations of total solids, oil and grease, chemical oxygen demand (COD), and biological oxygen demand (BOD) [6].

The biological treatment depends enormously on consortium of microorganism's activities, which operate the organic substances present in the POME as supplements and eventually degrade these organic matters into simple by-product such as methane, carbon dioxide and hydrogen supplied, and water. The biological treatment process requires large pond to hold the POME in place for the effective biodegradation, which regularly takes a few days relying upon the sort and native of the microorganisms [1]. Besides, so as to enhance the effectiveness of this medication process, powerful mono or combined culture of feasible fungi and bacteria in biodegradation treatment of POME waste. Therefore, the challenge of converting POME into an environmentally friendly waste demanded an efficient treatment and effective disposal technique.

A variety of microorganisms have been investigated to be capable of biodegrading oil wastewater with high profits. Anaerobic and aerobic treatments are the great and capable biological methods for POME treatment. However, the suspended and colloidal components are neither effectively decomposed biologically nor by other conventional means because their floating on the surface of the wastewater has an impact on the microbial cycle [7]. It is the major problem to cause failure of treatment system. Particularly, the major component in biological digestion, microorganism plays an important role and core factor of the system to control reactor performance and stability.

This research intended to investigate the viability of microorganisms present in POME under conditions for treatment. The process is investigated in terms of COD removal and decolorization (ADMI) with pH tolerance. This experimental work carried out on the activated sludge processes would give some accepting treatment of the microbial behaviour as well as the substrate removal performances and evaluation of the application of an existing palm oil waste on digestibility of POME condition.

## 2. Materials and Methods

### 2.1. Experimental Setup

**Figure 1:**
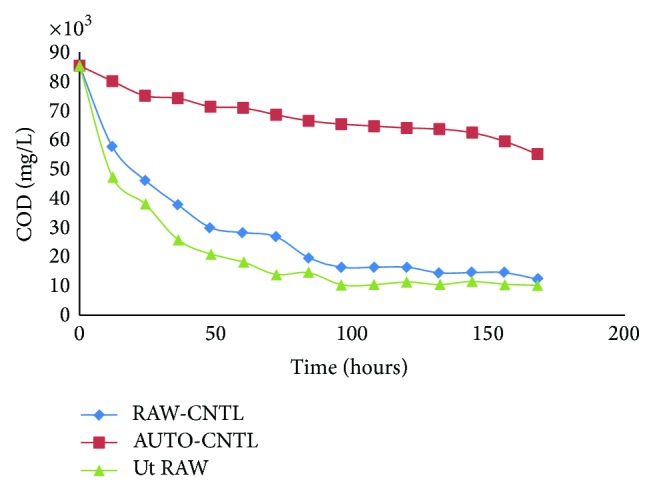
COD (mg/L) of autoclaved (AUTO-CNTL) and unautoclaved (RAW-CNTL) samples digested with inoculated multimicroorganism (Ut RAW). The control sample was not inoculated (RAW-CNTL).

**Figure 2:**
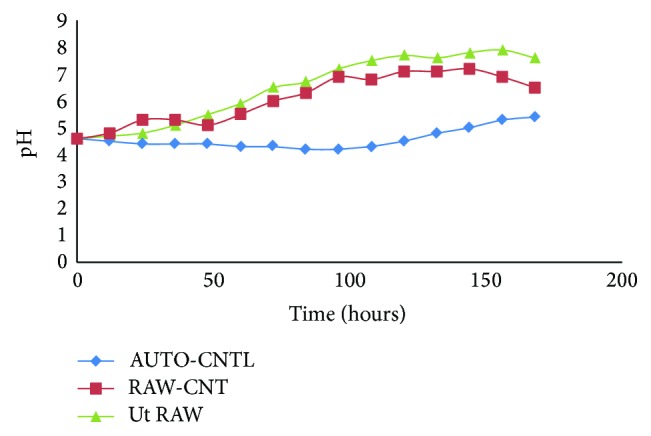
pH of autoclaved (AUT-CNTL) and unautoclaved samples digested with inoculated multimicroorganism (Ut RAW). The control sample was not inoculated (RAW-CNT).

**Figure 3:**
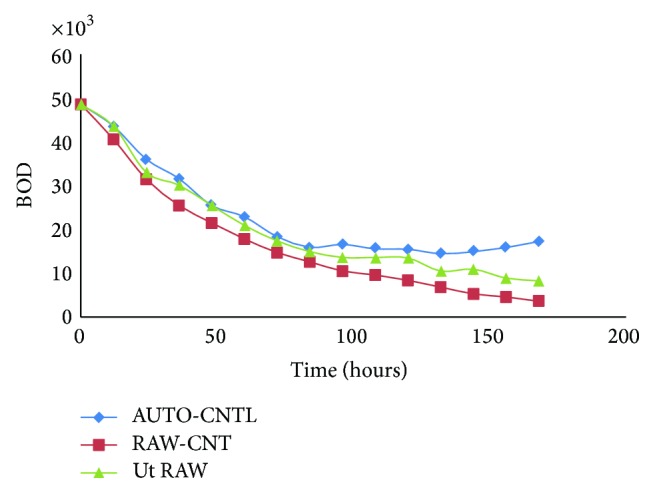
BOD (mg/L) of autoclaved (AUTO-CNTL) and unautoclaved samples digested with inoculated multimicroorganism (Ut RAW). The control sample was not inoculated (RAW-CNT).

**Figure 4:**
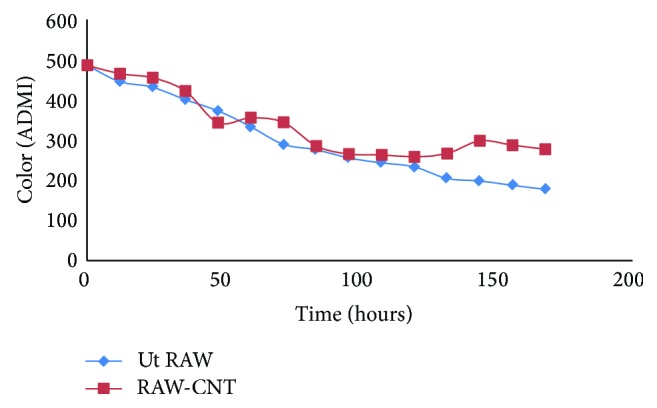
Color (ADMI) of autoclaved and unautoclaved samples digested with inoculated multimicroorganism (Ut RAW). The control sample was not inoculated (RAW-CNT).

Experiment was conducted in 250 mL Erlenmeyer flasks that contained 100 mL of POME that was covered with cotton gauze stopper. To study the effectiveness of microorganism's type on POME treatment, the batch tests were conducted under mesophilic condition. The medium was inoculated with inoculums culture (10% v/v) containing approximately 10^6^ cfu/mL of mixture of the isolated microorganisms (bacteria and fungi) in separate flasks and incubated at 37°C on an incubator shaker (150 rpm) for 7 days. The flasks containing the POME samples were autoclaved at 121°C and 15 psi for 15 min to produce the autoclaved samples. Both the autoclaved and raw flasks were inoculated with 5% (v/v) of the prepared inoculums. Three parameters were investigated in this study, whereas the effects of BOD, COD, pH, and ADMI were investigated (Figures 1, 2, 3, and 4). The incubation period depends upon the microorganisms and inoculum size of the colony used. The culture was transferred to POME medium for preparation of seed culture.

### 2.2. Palm Oil Mill Effluent (POME)

Raw POME sample was collected by grabbing a sample directly from the inlet of a waste stabilization pond which discharged from stone and sand filtration from local industry in Johor Bahru province in Malaysia. The sampling point was at least 100–150 cm deep of POME pond [8]. Sample was collected in clean containers, was directly transferred to the Department of Biotechnology and Medical Engineering Laboratory, University Technology Malaysia (UTM), and was stored at 4.0°C in the laboratory cold room for further treatments. The effluent was prefiltered by means of simple depth filtration to remove the coarse solid found in the suspension and experimental procedure for effect of microbe growth on the POME reduction. Raw POME has a dark brown color and pH of 4.2–4.5. The chemical characteristics of the POME are given in Table 1.

### 2.3. Enumeration of Total Heterotrophic Bacteria and Fungi

The populations of microorganisms in the samples were enumerated using by viable count procedures [9]. POME samples were collected from open pond system which is the most popular treatment method and is utilized by more than 85% of mills [10]. Serial dilution starts with a primary sample. About 0.1 mL of POME sample was serially diluted in sterile distilled/deionized water and aliquots of the dilutions were ascetically plated into the media (nutrient agar and nutrient broth, resp.). The agar plates were incubated at 37°C for 24–48 hours to enumerate the aerobe and facultative bacteria and the fungi culture plates were incubated and inverted at 37°C for 3–7 days. After incubation, the colonies that grew on the medium were counted and expressed as colony morphological and physiological characteristics. Microbial colonies were isolated and transferred into fresh cultures and preserved for further analysis. The dilution/isolation streaking procedure involves the dilution of bacteria by streaking them over the agar surface to obtain isolated cells which will subsequently grow into isolated colonies.

### 2.4. Identification of Microbial Isolates

A total of 70 facultative aerobe colonies were isolated from POME and cultured on two different media, nutrient agar, and nutrient broth. It is found that potential of treatment of POME was that ten of the colonies had the ability to reduce the COD and decolorization. Characterization and identification of aerobes to genus level using biochemical tests based on Tables 3 and 4. The characterization of POME microorganisms that are subcultured were identified by comparing their characteristics with biochemical tests. Identification of fungi was based on microscopic morphology and cultural characteristics and the bacteria isolates that were characterized based on biochemical test [11]. The bacteria isolates were identified using biochemical test. The derived characteristics were compared with those known using manual of determinative bacteriology and isolated bacteria were compared with the culture characteristics. Fungi identification was based on the macroscopic and microscopic morphology [12]. For the microscopic morphology, a drop of ethanol was placed on a clean slide with the aid of a sterile needle, a fragment of the pure culture was transferred into the ethanol on the slide, and a drop of lacto-phenol blue stain was added and the ethanol was allowed to evaporate. The observations with microscopic and macroscopic characteristics were compared, respectively. The bacteria isolated from the POME included* Aspergillus niger, Aspergillus flavus*,* Mucor* sp.,* Fusarium* sp.,* Penicillium* sp.,* Staphylococcus aureus, *and* Bacillus* sp. inoculated into the autoclaved sample and then inoculated sample based on isolated microorganisms was incubated (10^6^ cfu/mL) at 37°C and agitated at 150 rpm.

### 2.5. Analytical Methods

The pellet from the centrifugation of POME was used to determine the growth of the bacterial consortium. Two mL of distilled water was added into the pellet and was mixed homogenously using pipette. They were then transferred into the cuvette and the growth was measured using spectrophotometer at 600 nm wavelength. To separate the fungal biomass and the liquid medium, the whole fungi culture was centrifuged at 4,000 rpm for 15 min at 4°C. Analytical parameters of POME were analyses based on the methods developed and modified by the Department of Environment of Malaysia, DOE [13], such as COD (digestion method), BOD (biochemical oxygen demand), and the color (ADMI unit), which were determined according to the APHA method [14]. The pH was measured using the Sartorius PB-10 pH meter. All experiments were conducted in triplicates.

#### 2.5.1. Determination of COD Removal

The analysis of COD was carried out according to the oxygen equivalent of the organic material in wastewater [15]. Preliminary determination of COD usually relies on the amount of organic pollutants found in water and wastewater. The remained supernatants from each of the samples taken from three-hour intervals were used to determine the COD. One mL of the supernatant of each treated sample was transferred into COD vial and it was diluted with 0.9 mL distilled water (COD 0–1500 ppm range) and the mixture was mixed gently. The vial was placed in the preheated COD reactor at 150°C for 2 hours. The percentage of COD removal was determined by cooling it at room temperature for 1 hour and determined by the spectrophotometry method. The absorbance was read at 620 nm (COD 0–1500 ppm range using HACH program). For the blank, deionised water was used for the background subtraction. All analyses were run in triplicates [16].

#### 2.5.2. ADMI (American Dye Manufacturers' Institute Colour)

The color was determined by the American Dye Manufacturers Institute (ADMI) method; the light scan absorbance was from 400 to 700 nm using Shimadzu UV-160A, UV-visible recording spectrophotometer [17]. The supernatant was also used to determine the decolorization of POME. Thus, 0.1 mL of the supernatant was diluted with 3.9 mL of phosphate buffer. They were mixed homogenously prior to transfer into the cuvette to be quantified of their degree of decolorization. The quantification was done by measuring the reduction of optical density (OD) at 471 nm.

#### 2.5.3. Determination of Biochemical Oxygen Demand

Biochemical oxygen demand (BOD) test is a widely used parameter for evaluating the ability of naturally occurring microorganisms to digest organic matter [18], in 3–7 days incubation at 37°C by analyzing the depletion of oxygen inside the POME. BOD is the most commonly used parameter for determining the oxygen demand on the receiving water of a municipal or industrial discharge. BOD can also be used to evaluate the efficiency of treatment processes and is an indirect measure of biodegradable organic compounds in water. This experiment was done by dilution method. A measured amount of the biodegradation sample was poured into the 300 mL BOD standard bottle.

## 3. Results and Discussions

The result from this study shows that the microbial species isolated are similar to those found in local POME. However,* Pseudomonas* species,* Staphylococcus* aureus, and* Bacillus* species, which are* Bacillus* sp.,* Pseudomonas* sp., and* Staphylococcus* sp., are lipase producing organisms associated with pathogenicity [19]. Spores make POME microbial species to be inactive and highly resistant to lethal effect of boiling, dry heating, and ultra violet radiation [20]. POME is potential habitat for thermophilic lipolytic bacteria [21, 22]. Identified* Aspergillus* sp. are fungal species associated with lipase production. Lipase accelerates in hydrolysis of lipid causing consequent breakdown into fatty acid and alcohol [23, 24].* Aspergillus niger* and* Aspergillus flavus* have been distinguished for their ability to endure oily wastewater such as POME. The presence of* Penicillium* sp.,* Fusarium* sp., and* Mucor* sp. in the POME shows that these fungi are able to survive in hostile environment [21, 22, 25]. According to previous reports, these organisms can degrade oily wastewater effectively.

The value of pH recorded in through this study is lower, that is, more acidic than the Ohimain et al. [26]. The low pH of the POME indicates that it is acidic and should be treated to reach 7–7.5 pH as indication level of plant compatible. The acidic character of POME may have been influenced by the corrosion of iron used in processing. When the POME is discharged into the soil or stream, it affects nutrient availability of the nearby plants [27]; in addition, most plants grow within a pH range of 6.5–7.5 [28]. Decolorization is one of the vital parameters in POME quality assessment and indicates the physical and biological processes prevailing in the POME; it points to the degree of pollution in wastewater [29, 30].

The major predominate microorganisms in POME have been variously mobilized for the treatment of the wastewater [31–34]. During degradation, oil and grease in the POME are broken down and mineralized [35]. The bacteria found in POME are useful during upflow anaerobic sludge blanket system treatment [36]. The oily environment may provide a good environment for lipolytic microorganisms to flourish due to the unrecovered oil present in the effluent. However, the microbial content of POME is a good indicator of biodegradability of wastewater and it could also be used in bioremediation of hydrocarbon from crude oil spills. However, since most of these organisms are spore formers, it helps them to survive the harsh environmental conditions of POME such as acidity [37, 38]. Moreover, the dark brown color of palm oil mill effluent is composed of organic compounds such as anthocyanin and carotene pigment that were extracted from fresh fruit bunches in the sterilization process [39]. Furthermore, it included polyphenol compounds, tannin, polyalcohol, and melanoidin [39, 40].

### 3.1. COD Concentrations of Digested Samples

The COD removal for the biodegradation of POME samples is depicted in Figure 1. In general there was gradual decrease in the COD concentrations of the raw POME sample including the inoculated multimicroorganism which was selected in this study and the control. The control sample tendency was observed for the biodegradation which was used as the noninoculum under controlled which is suspected to be undergoing some biochemical synthesis in 7 Days. Furthermore, it was observed that the removal of COD in the autoclave sample was very close to the raw sample and this may suggest inoculated sample with isolated microorganism in the raw sample. In addition, the activities of local microorganisms may be used to describe the degradation of raw POME with high degradation of POME.

### 3.2. Change in pH of Digested Samples

The pH of the entire biodegradation samples increased from acidity towards alkalinity; processes increase alkalinity though at different rate and dimensions (Figure 2). The control raw (RAW CNTL) sample and those inoculated with selected microorganism, respectively, reached neutral point at the end of the period and One important point to note is that treatment should reach to high standard of pH of POME for releasing through the environment. At the other end is a point of maximum expected in COD and BOD reduction with proper fungi and bacteria in biodegradation of POME. A pH of 7.6 could vastly increase the benefits, since the treatment of the POME is for plant reutilization and discharge into river streams with less effect on the environment. Furthermore, the pH of the autoclaved samples is the case in the study which was normally below the pH of the unautoclaved samples except for sample with microorganism treatment. Moreover, close relationship was observed in the trend of pH changes between the raw and autoclaved POME sample inoculated with privilege of selected microorganism; however, treatment could accelerate the biodegradation in process.

### 3.3. Changes in Biochemical Oxygen Demand (BOD) of Samples

The changes in the biochemical oxygen demand (BOD) of the samples were checked out for seven days (164 hours) in the BOD incubator (Figure 3). The concentrations of the BOD generally decreased with numbers of days for all the samples inoculated with multimicroorganisms as well as the control sample but treatment shows stability in final stage. This trend is positive to the biodegradation treatment process of high strength wastewater such as POME, because the decrease in the BOD indicates the reduction in the environmental organic load on the receiving water stream.

### 3.4. Changes in ADMI of Samples

The color was determined by the American Dye Manufacturers Institute (ADMI) method. The changes in the color of the samples were checked out for seven days (164 hours) based on the ADMI method at 471 nm (Figure 4). The concentrations of the color generally decreased with numbers of days for all the samples inoculated with multimicroorganisms as well as the control sample but treatment shows stability in final stage, whereas there are fluctuation for control group which indicates contradiction in metabolites of microorganism system (growth between 60–71 hours and 132 hours). This treatment shows positive tendency in biodegradation treatment of high strength wastewater such as POME, because the decrease in the color indicates the reduction in the environmental organic as a control application. Figure 4 shows the effect of color removal efficiency on POME treatment. It is really an indication that colorization is a vital parameter for determining biodegradation which directly signifies the metabolic status of probably the most delicate microbe group within the biodegradation system. The color reduction denotes the process stabilization.

### 3.5. Identification of the Efficient COD and BOD Removal

The COD and BOD reduction performance was monitored each 12 hours in batch system consisting of multibacteria isolated from POME wastewater.  Table 3 shows the result for COD and ADMI values after treatment by using single bacteria in batch system each 12 hours in 7 days.

The adaptation of the mesophilic microorganism operating condition at 37°C led to a stable process that is significantly different from that previously found at 37°C (Table 2). This new process allows for a conversion of organic matter into the final treatment. This could be attributed to the rapid development of biodegradation wastewater that was originally present in the mesophilic sludge to become dominant under the new degradation conditions based on isolated local microorganisms. A total of 10 aerobe colonies fungi and bacteria were identified in POME. Seven fungal and five bacterial species were persistent. The persistent fungi were* Aspergillus niger, Aspergillus flavus, Aspergillus fumigates, Fusarium, Penicillium, Mucor, *and* Candida* and bacteria were* Pseudomonas, Micrococcus, Staphylococcus aureus*, and* Bacillus.* Comparatively more fungi were identified on palm fruits in POME sample. This could be due to the fact that a substantial amount of these organisms may be eliminated from palm fruits when processing into the oil or contaminated after discharge into pond. High population of fungi in the POME may be associated with contaminations from poor sanitation in the mills. Moreover, it may also be due to the treatment process and the existing environmental conditions in the mills. The microbial species found in POME has the potential of degrading hydrocarbon in the wastewater. Biodegradation is associated with the saprophytic ability of fungi to grow on and degrade carbon sources in industrial effluents and reduce factor which could influence the environment. In this study, the acidic tendency of POME treatment (Table 2) may affect nutrient availability of the nearby plants which pasture from discharge of POME in river; final pH reached approximately 7.6 (Ut RAW) in appropriate range rather than RAW-CNT and AUTO-CNTL (6.5 and 5.4, resp.) (Figure 2); most plants grow and do better within a pH range of 6.5–7.5. BOD is an important parameter in POME quality measurement and indicates the physical and biological processes surpass in the POME biodegradation; it indicates the degree of pollution in wastewater, and BOD value obtained during the study was in the range of 155000–168000 mg/L (Figure 3). The COD was an important aspect to estimate the organic content in POME. The effect of different oxygen concentration on the reduction of COD was depicted in Figure 1. It implies that the population of microorganisms plays a vital role in the process of reduction of COD, thus* purged *wastewater. Figure 1 showed that the number of bacteria increases and at the same time causes the reduction in COD concentration of POME, approximately 12000 mg/L in 168 hours of treatment. Figure 4 presents the colorization which shows positive tendency in biodegradation treatment of high strength POME; color measurement indicates the reduction in the environmental organic 486 to 180 ADMI. The result showed that all the samples collected from various mills have similar microbial species. The bacteria isolated from the POME include* Aspergillus niger, Aspergillus flavus*,* Mucor* sp.,* Fusarium* sp.,* Penicillium* sp.,* Staphylococcus aureus, *and* Bacillus* species inoculate into the autoclaved sample (10^6^ cfu/mL) incubated in flask in stable incubator.

### 3.6. Optimum Physicochemical Conditions for Growth

The growth of the potential isolated* Aspergillus fumigatus* and* Aspergillus niger* were determined at different temperatures. Substantial growth was observed at temperatures 35–37°C. In the case of growth at different condition with mixture of local microorganism, the optimum temperature had maximum growth at 37°C and agitated at 150 rpm.

## 4. Conclusion

Environmental issues have been gradually increased to becoming one of the most important in economic activities. In addition, the physicochemical conditions of POME from palm oil mills was studied. The following parameters: pH, ADMI, BOD, and COD, were analysed using standard methods. The synergistic effect of bacteria and fungi, for POME treatment, brings about enhanced performance for effective biodegradation. BOD, COD, and OD values obtained during the study were in the range of 155000–168000 mg/L, 12000 mg/L, and 180 ADMI, respectively (Figures 1, 3, and 4) and pH was tending toward neutral point (Figure 2) contrary to previous studies (Table 2). The viability of using isolated microorganisms for effective biodegradation of POME has been investigated in this study and it can be deduced that the digestion of POME samples based on local isolated fungi and bacteria. This result indicates the potential of isolating of indigenous microorganism that will be effective in the degradation POME; on the other hand, the isolated microorganism requires nutrient expansion for the effective biodegradation of POME. Due to the high concentrations of BOD and COD in the oil palm processing effluents, discharging it into the environment could cause pollution; hence, it is preferable to recycle rather than discharging it. By improving environmental condition of living microorganisms in a biodegradation process, substrate-related factors could be utilized in bringing up a flourishing achievement in aerobic waste degradation process. The addition of functional inoculum and selected high effective indigenous microorganisms is expected to save the POME degradation time and cost for reduction of BOD, COD, and color.

## Figures and Tables

**Table 1:** Chemical characteristics of palm oil mill effluent (POME) used in this study.

Parameter	Concentration (mg/L)
Biochemical oxygen demand	(BOD) 23,400–52,100
Chemical oxygen demand	(COD) 80,100–95,000
Total carbohydrate	17,000–19,000
Total nitrogen	850–930
Ammonium nitrogen	24–31
Total phosphorus	96–120
Phosphate	15.2–20.6
Oil	8,500–11,000
Total solids	36,000–43,000
Suspended solids (SS)	9,400–12,500
Ash	4,200–4,600

**Table 2:** Physicochemical properties of palm oil milling effluents.

Parameters	This study (mean ± standard error)	[22]	[26]
pH	4.63–4.70	5.213–6.357	6.56 ± 0.05
DO, mg/L	2.355–3.766	2.567–4.127	4.69 ± 0.00
COD, mg/L	85340–11835	1231–2422	1806.33 ± 7.12
BOD, mg/L	48555–48994	254–1541	382.93 ± 0.89

**Table 3:** Biochemical test of bacteria isolates of POME.

Organisms	Cultural characteristics	Gram reaction	Motility	Oxidase	Catalase	Citrate	Coagulase	Urease	Indole
*Pseudomonas* species	Mucoid colonies with umbonate elevation	Negative rod	+	+	+	+	−	−	−

*Micrococcus* species	Circular, pinhead colonies which are convex with entire margins. Colonies produces a bright yellow, nondiffusable pigment	positive cocci	−	+	+	−	−	−	−

*Staphylococcus* aureus	Circular, pinhead colonies which are convex with entire margins. The colonies are golden-brown to whitish in color	positive cocci	−	−	+	−	+	−	−

*Bacillus* species	Dry, flat, and irregular, with lobate margins	Positive rod	+	+	+	+	−	−	−

**Table 4:** Microscopic morphology and cultural characteristics of fungi isolates of POME.

Organisms	Type of organisms	Microscopic morphology	Macroscopic morphology
*Aspergillus niger *	Filamentous mold	Presence of septate hyphae, long and smooth conidiophores, and long unbranded sporangiospores with large, round head.	Creamy to brownish-black mycelium with dark spores and often appears golden on the reverse side

*Aspergillus flavus *	Filamentous mold	Presence of septate hyphae and colourless and rough conidiophores with swollen vesicles.	A greenish-yellow colour with a creamy edge that appears golden in the reverse of the septate

*Aspergillus fumigatus *	Filamentous mold	Presence of rough conidiophore, with uni/biseriate phialides whose vesicle is round with radiate head. Brownish sclerotia were also observed.	Presence of blue-green to yellow coloration from surface

*Fusarium* sp.	Filamentous mold	Presence of dark pigment of micro- and macroconidiophores and it is spherical in shape.	Presence of sickle shaped macroconidia that is yellow to purple in color

*Penicillium* sp.	Filamentous mold	Presence of red pigment with edges surrounded by whitish margin. Also the conidiophores are branched. Septate and fruity mycelium are observed	A bluish-green filament is seen which changes to powdery greenish brown. Phial-spores arrangement

*Mucor* species	Filamentous mold	Presence of visible spore and short sporangiospores with nonseptate hyphae	A slimy colonies texture with dark pigmented spores

*Candida* species	Ovoid sphere yeast-like	Single clusters of blastoconidia which is round and elongated. Long branched pseudohyphas were also observed.	Creamy to yellowish colonies with smooth, pasty, glistening, or dry
